# Cochlear implantation in the elderly: surgical and hearing outcomes

**DOI:** 10.1186/1471-2482-13-S2-S1

**Published:** 2013-10-08

**Authors:** Benatti Alice, Montino Silvia, Girasoli Laura, Trevisi Patrizia, Bovo Roberto

**Affiliations:** 1 ENT Department, University Hospital of Padua, Italy

## Abstract

**Background:**

At the present time, 50 to 60% of the population above 70 years of age suffers from a hearing impairment and from 0.6 to 1.1% has a severe to profound loss, which cannot benefit from an hearing aid. Moreover, it is expected that this prevalence will grow by more than two-fold in the next 40 years. There is strong evidence that hearing loss in older adults is associated with both cognitive load and social isolation, which in turn, are associated with cognitive and physical functioning. Cochlear implant (CI) dramatically improves sound audibility and speech understanding. The aim of this paper was to analyze outcome and complications of CI treatment in elderly patients.

**Methods:**

A retrospective study on 17 patients, aged at implantation between 65 and 79 years (mean = 70.47 ± 3.94), unilaterally implanted for severe to profound bilateral hearing loss. The following data were statistically evaluated: pre-implant pure-tone threshold and tests of speech recognition, both with hearing aid that without; post-implant threshold and speech perception with CI off and on. Moreover, statistical correlations of PTA improvement between two age groups (65 to 70 and over 70 years) were carried out.

**Results:**

Mean PTA improved from 111.25 (± 17.51) (pre-implant) to 43.81 (± 9.27) (post-implant); and the mean SRT improved from 90 dB to 65 dB. Moreover there was no statistical difference in PTA improvement between the two age groups (65 to 70 and over 70 years). No severe per- or post-operative surgical complications were noted.

**Discussion:**

In the elderly, CI is a safe procedure that significantly improves hearing threshold (p < 0.00001) and speech perception (p < 0.01). Support of family and professionals, as well as duration of deafness and pre-implant scores greatly influence the results of rehabilitation and its perceived benefit. CI should not be denied in older individuals who are otherwise in good health.

## Content

### Background

At the present time, 50 to 60% of the population above 70 years of age suffers from a hearing impairment and from 0.6 to 1.1% [1,2] has a severe to profound loss, which cannot benefit from an hearing aid. Moreover, it is expected that this prevalence will grow by more than two-fold in the next 40 years.

**Figure 1:**
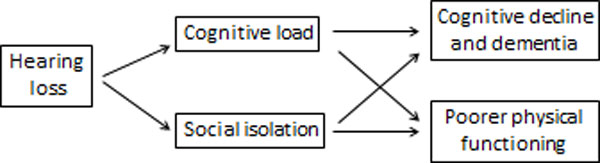
**Conceptual model of the association of hearing loss with cognitive and physical functioning in older adults (from F. Lin, 2012)**[3]

There is strong evidence that hearing loss in older adults is associated with both cognitive load and social isolation, which in turn, are associated with cognitive and physical functioning (Figure 1) [3].

Individuals with a mild, moderate and severe hearing loss had a two-, three-, and five-fold increased risk of developing incident dementia, respectively, compared to normal-hearing individuals [3]. Moreover, analyses of the association of hearing loss with self-reported falls demonstrated that a 10 dB increase in hearing loss was associated with a 1.4 fold increased odds of having a fall. A 25 dB hearing loss was associated with a nearly three-fold increased odds of a fall over the preceding year. These results were substantively unchanged after adjusting for demographic and cardiovascular risk factors as well as vestibular balance function [4].

**Figure 2:**
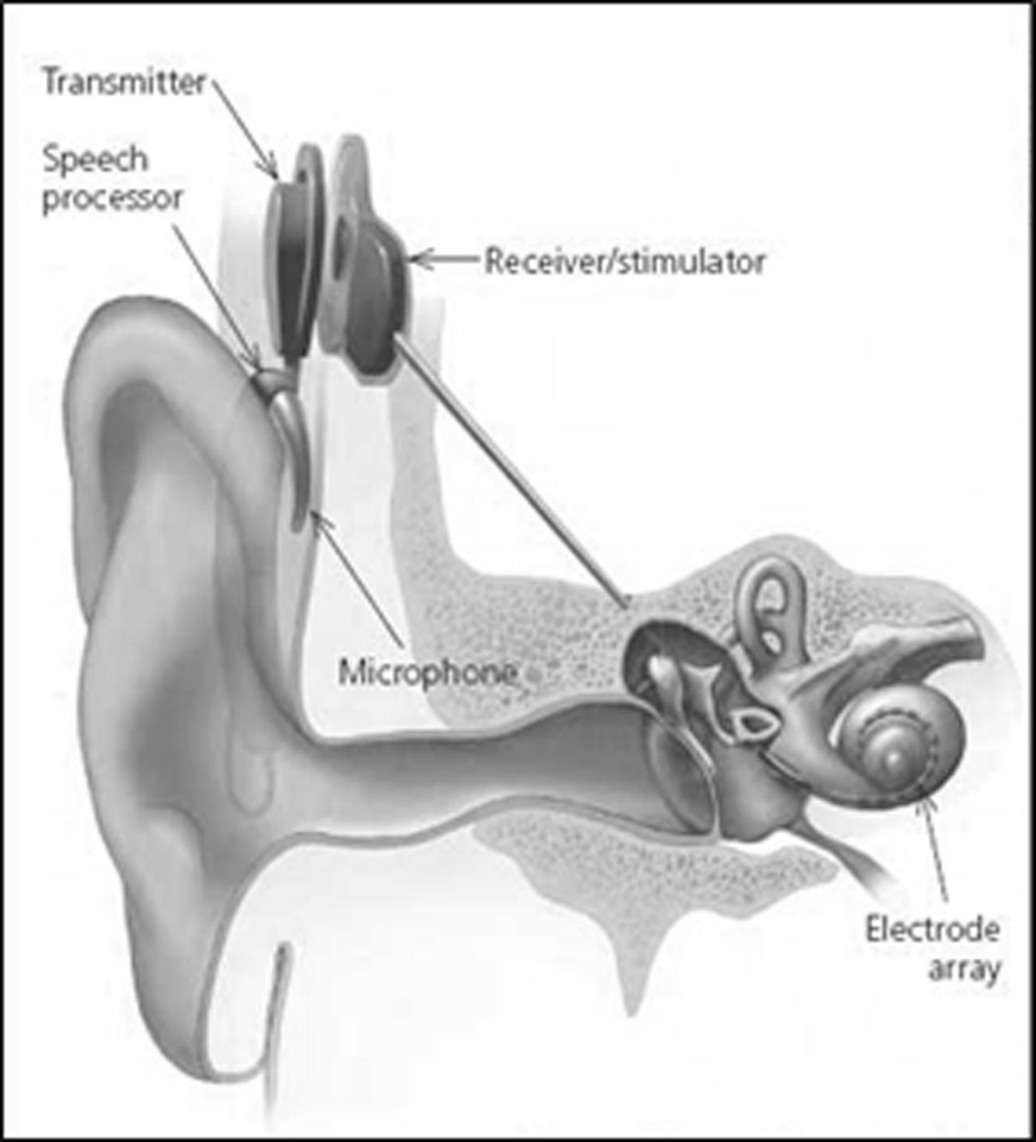
External and internal parts of a cochlear implant

It is well demonstrated that cochlear implant (CI) dramatically improves sound audibility and speech understanding for the elderly patients, similarly to the young implanted patients. CI was a significant surgical innovation in the 20th century and represented the first artificial sensory organ applied in clinical medicine. It is a partially implanted electronic device that can evoke acoustic sensations by electrically stimulating the inner ear and is constituted by an external portion, that usually sits behind the ear and an internal portion surgically placed under the skin. The external components include a microphone connected to a speech processor that selects and arranges sounds picked up by the microphone. This is connected to a transmitter coil, worn on the side of the head, which transmits data to an internal receiver coil placed under the skin. The received data are delivered to an array of electrodes that are surgically implanted within the cochlea. The primary neural targets of the electrodes are the spiral ganglion cells which innervate fibers of the auditory nerve. When the electrodes are activated by the signal, they send a current along the auditory nerve and auditory pathways to the auditory cortex. (Figure 2)

It was previously thought that CI in the elderly may not be beneficial because of age-related degeneration of both the central and peripheral auditory systems, surgical risk, and overall cost to benefit ratio. However, recent studies have shown that this procedure improves auditory performance, is well tolerated even in the most elderly, enhances self-confidence, reduces in most cases tinnitus and stress and increases the health-related quality of life. The risk of anesthetic and surgical complications remains low provided that a through multidisciplinary evaluation is performed before the procedure. The cost-effectiveness still remains acceptable, including patients over 70 [5] because even if healthcare costs are high, the savings in terms of indirect costs and quality of life are important. Among patients with pre-implant severe tinnitus, a partial or total tinnitus reduction was observed in 70% of cases [6].

### Methods

This is a retrospective study of 17 consecutive post-lingual, profoundly hearing impaired elderly adults selected among the overall 282 patients who were implanted at Padua ENT-Ear Surgery Department between May 2010 and February 2013. Selection criteria were age ≥ 65 yrs at surgery and unilateral implantation. Pre-implant evaluation consisted of pure-tone audiometry and tests of speech recognition, both with hearing aid that without. Post-implant evaluation included the same tests with CI off and on, carried out with free field stimulation in a sound proof booth. Threshold evaluation were conducted by using pure-tone average (PTA), that is the mean of the air-conduction thresholds at 500, 1000 and 2000 Hz. On the other hand, in the analyses of speech perception we considered the Speech Detection Threshold (SDT) and Speech Recognition Threshold (SRT). SDT corresponds to the value of sound intensity at which the verbal message is not understood but perceived as generic sound, therefore with a percentage of intelligibility of 0%. The SRT indicates the level of intensity at which the patient correctly repeats 50% of the words.

Surgical outcome looked at the presence of any medical or surgical complication related to the implant surgery or to the age of these patients.

### Results

Our sample is composed by 17 patients (9 F - 8 M) aged at implantation between 65 and 79 years (mean = 70.47 ± 3.94), that represents a 6.0% of our cochlear implantations during the considered period. Based on our experience, with respect to younger CI-recipients, this group of elderly had a higher incidence of associated comorbidities such as arterial hypertension, cardio-vascular diseases, usage of anticoagulants. Despite this, no surgical events or complications with the anesthesia were observed and there was no need for additional intensive postoperative care.

The duration of hearing loss ranged from 1 to 50 years: 8 patients were deaf from ≤15 years and 9 were deaf from >15 years. The etiology was unknown in the majority of cases (52.9%), while the most frequent known cause was otosclerosis (29.4%). (Table 1)

**Table 1:** General information of implanted patients

Patient	Sex	Age implant (years)	Date implant	Side	Aetiology	Duration hearing loss (years)
1	M	66	28/05/2010	L	otosclerosis	15
2	F	72	05/11/2010	L	unknown	10
3	M	70	07/12/2010	R	unknown	15
4	M	68	18/03/2011	R	trauma	1
5	F	72	05/04/2011	L	unknown	9
6	F	77	19/05/2011	L	unknown	45
7	F	72	22/07/2011	R	unknown	30
8	M	71	14/09/2011	R	otosclerosis, trauma	8
9	F	73	02/11/2011	L	unknown	30
10	F	67	29/02/2012	L	neurinoma, trauma	30
11	M	72	13/03/2012	L	unknown	15
12	F	66	25/07/2012	L	otosclerosis	40
13	F	66	07/09/2012	L	otosclerosis	15
14	M	69	17/10/2012	L	otosclerosis	30
15	M	79	05/10/2012	R	unknown	50
16	M	65	08/02/2013	R	streptomycin	50
17	F	73	27/02/2013	R	unknown	18

First examination occurred one month after initial switch-on and programming of the speech processor ("activation"), followed by a second exam at 4 months, then at 7,11 and 15 months. Many of our elderly patients expressed initial disappointment, during the first switch-on session, mainly due to the novel sound quality provided through the electrical stimulation, but also due to the initial lack of benefit. However, all patients have adapted and over time becoming regular daily CI users.

**Figure 3:**
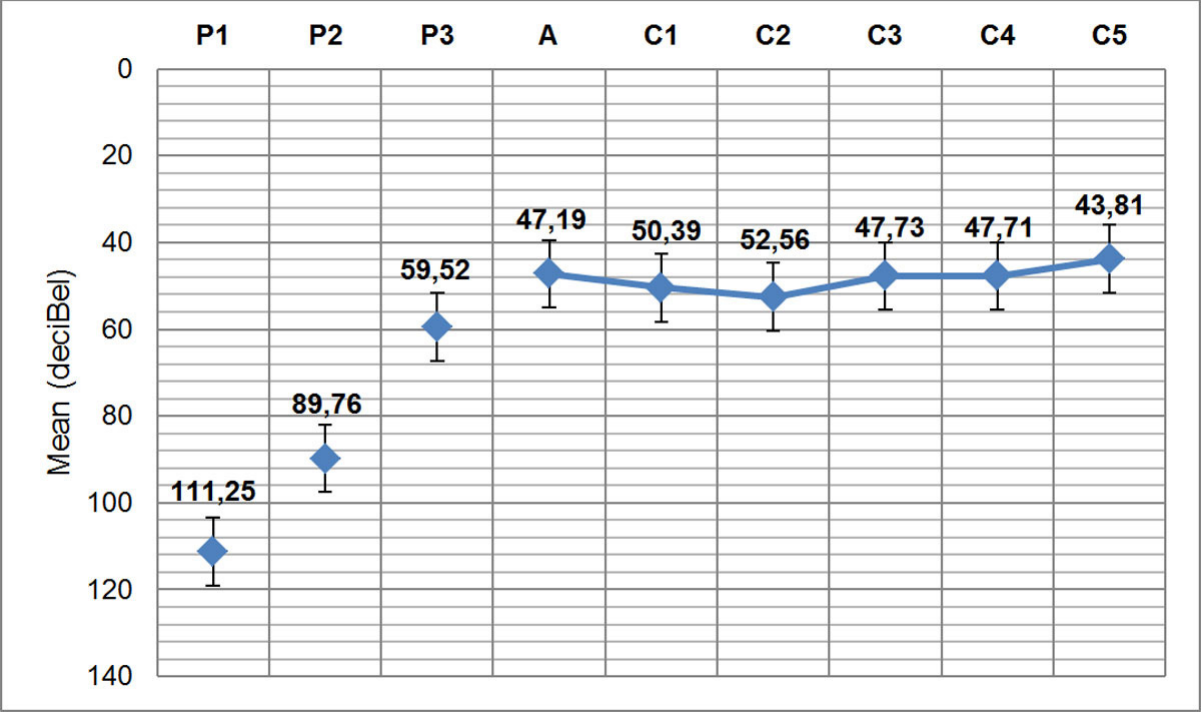
Pre- and post-operative PTA *(P1=pre-op, side implanted; P2=pre-op, free field without hearing aids; P3=pre-op, free field with hearing aids; A=post-op at activation; C1=post-op, 1st control; C2=post-op, 2nd control; C3=post-op, 3rd control; C4=post-op, 4th control; C5=post-op, 5th control)*

**Figure 4:**
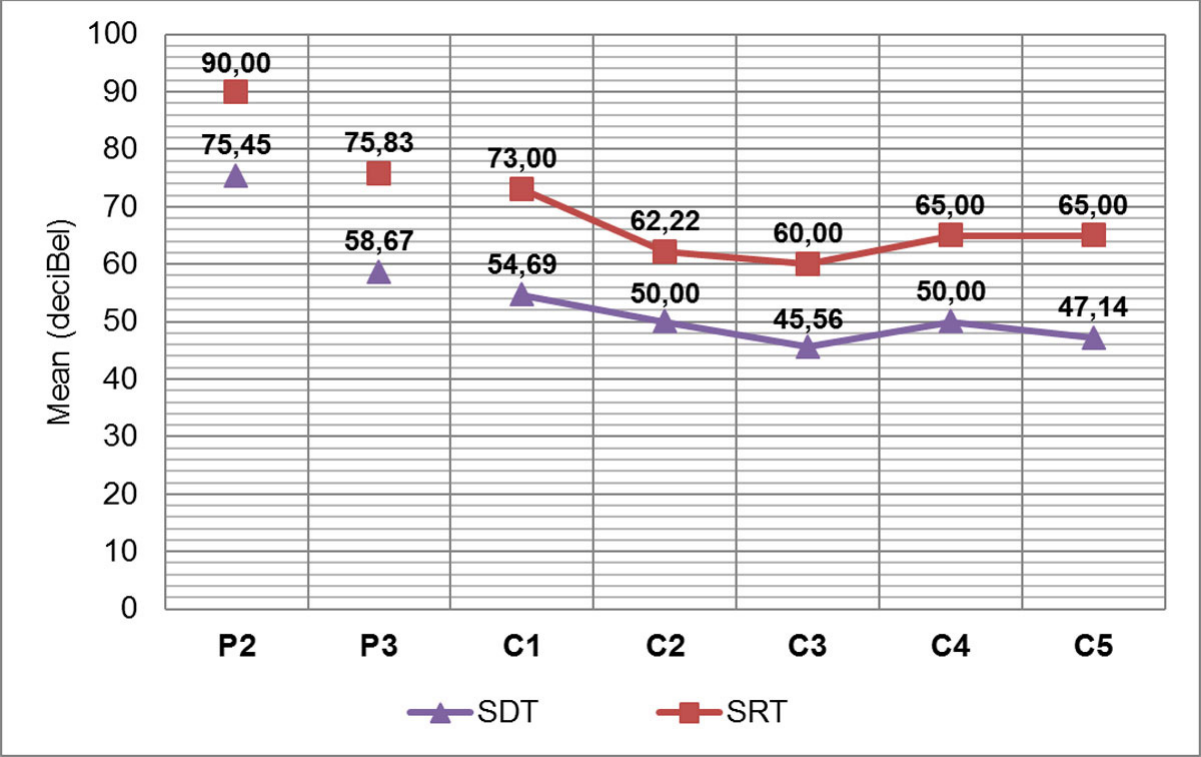
Pre- and post-implant mean speech detection threshold (SDT) and mean speech recognition threshold (SRT) *(P2=pre-op, free field without hearing aids; P3=pre-op, free field with hearing aids; C1=post-op, 1st control; C2=post-op, 2nd control; C3=post-op, 3rd control; C4=post-op, 4th control; C5=post-op, 5th control)*

PTA values were compared between pre- and post-implant exams demonstrating a significant improvement with CI (Figure 3). Also speech perception scores showed a significant improvement both in the detection threshold (SDT) that in perception threshold (SRT) (Figure 4).

These variables were analyzed using descriptive statistic and Student t test (P < .01).

Dizziness was the most common temporary complication and was observed in 4 cases (23.5%). It was not correlated with the pre-op morbidities of the affected patients and was resolved within a few days in post-op for all cases. One transient-incomplete facial nerve weakness was found 3 days post-surgery and completely resolved in a few days.

### Discussion

Our results demonstrated that CI in older adults is a safe procedure, which significantly improves hearing threshold (p < 0.01) and speech understanding (p < 0.001). In particular, mean PTA improved in our patients from 111.25 (± 17.51) (pre-implant) to 43.81 (± 9.27) (post-implant); and the mean SRT improved from 90 dB to 65 dB. Our data are similar to those reported by Skarzynsky et al. [7] and by Luntz et al. [8], who evaluated an elderly population of similar age with respect to our (mean age at implantation respectively of 67.2 yrs and 66.7 yrs). In fact, these authors observed mean word recognition scores increasing respectively from 17% (pre-implant) to 66% (post-implant) and from 18% to 60%. By comparing these results with those of children and young adults implanted at our center during the same period, we observed that the elderly need a longer rehabilitative period, but eventually all of them were regular CI users and reached similar good results. Major post-CI complications were not encountered in this cohort . Post-implantation vertigo was not as significant as might be expected in this age group.

In our experience, support of family and professionals, as well as duration of deafness and pre-implant scores greatly influence the results of rehabilitation and its perceived benefit. In conclusion, we strongly recommend that CI should not be denied in older individuals who are otherwise in good health.

## List of abbreviations used

CI: Cochlear Implant; PTA: Pure Tone Average; SDT: Speech Detection Threshold; SRT: Speech Recognition Threshold.

## Competing interests

The authors declare that they have no competing interests.

## Authors' contributions

BA: data collection and analysis. MS: data collection. GL: statistical analysis, manuscript draft and editing. TP: manuscript revision and study design. BR: manuscript revision and study design. All the authors had given final approval of the version to be published

## Authors' information

BA: MD, audiologist ENT Dep. of Padua. MS: speech pathologist ENT Dep. of Padua. GL: MD, resident ENT Dep. of Padua. TP: MD PhD, University researcher, audiologist ENT Dep. of Padua. BR: MD PhD, cochlear implant surgeon, audiologist ENT Dep. of Padua.

## Declarations

Funding for this article was by personal funds.

This article has been published as part of *BMC Surgery *Volume 13 Supplement 2, 2013: Proceedings from the 26th National Congress of the Italian Society of Geriatric Surgery. The full contents of the supplement are available online at http://www.biomedcentral.com/bmcsurg/supplements/13/S2
